# Assessment of acute radiation therapy-related cardiotoxicity by cardiovascular magnetic resonance

**DOI:** 10.1186/1532-429X-18-S1-P257

**Published:** 2016-01-27

**Authors:** Julius Traber, Robert Krempien, Jeanette Schulz-Menger, Florian von Knobelsdorff-Brenkenhoff

**Affiliations:** 1 Charité Medical Faculty and HELIOS clinics, Working group Cardiovascular MRI, Berlin, Germany; 2 Radiation Therapy, HELIOS clinics Berlin Buch, Berlin, Germany

## Background

Radiation therapy is an effective and broadly applied adjuvant in the treatment of many malignancies. Collateral radiation exposure to the heart can result in relevant cardiac disease, which might not be manifest until years after treatment. Aim of this study was to test whether cardiovascular magnetic resonance (CMR) identifies signs of early cardiac injury.

## Methods

We prospectively applied CMR at 1.5T before (a), at half-time (b) and after radiation therapy (c) in patients with different thoracic malignancies. Besides SSFP-based cine imaging for cardiac morphology and function, we performed pre- and post-contrast T1-mapping (MOLLI) as well as late gadolinium enhancement (LGE) imaging for tissue characterization. T1-times were assessed in a mid-ventricular short axis. LV Ejection Fraction (LVEF) and the partition coefficient λ were calculated.

## Results

**Figure 1:**
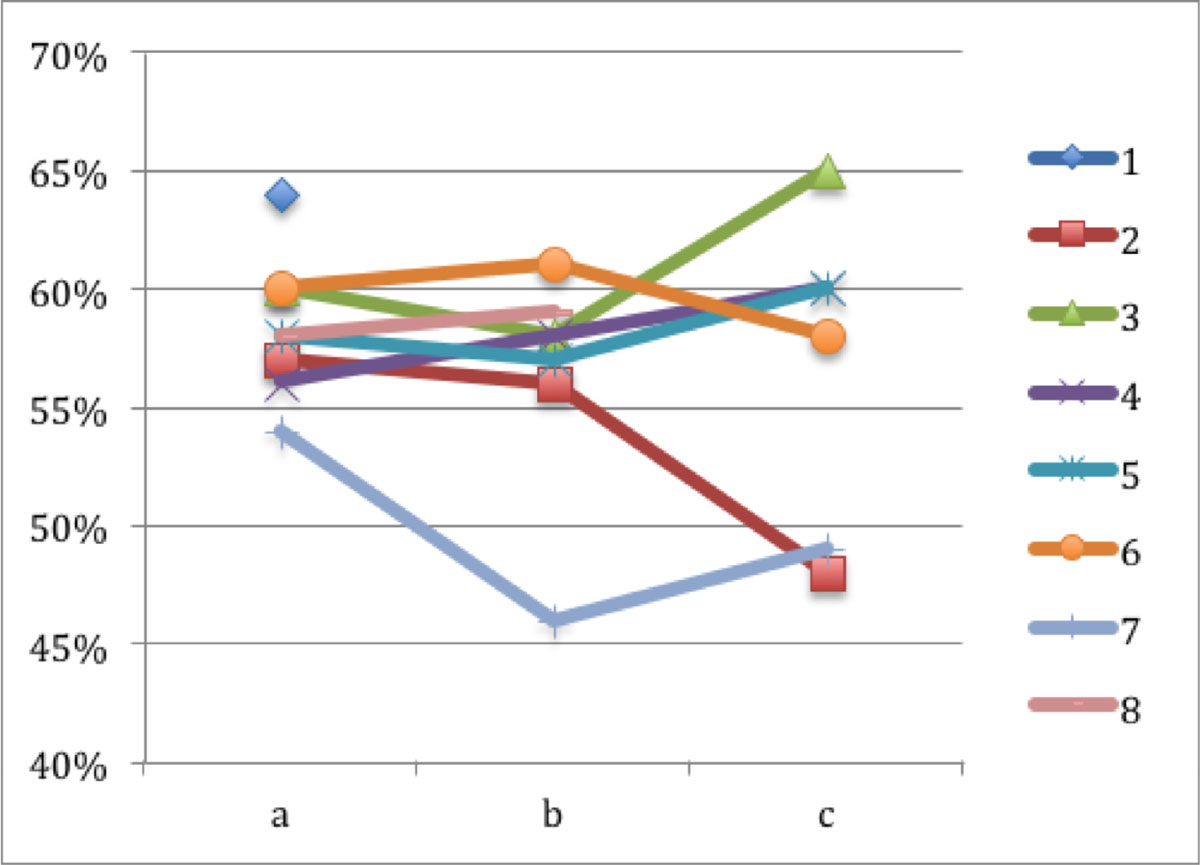
**Individual course of LV Ejection Fraction (y-axis) at baseline (a), at half-time (b) and after (c) radiation therapy (x-axis)**.

Ten patients (5 male, 51.4 ± 16.3years) were included. One patient was excluded due to an unknown metallic implant, one declined at baseline, one at half-time and one at after radiation therapy examination. Mean heart dose was 11.5 ± 9.6Gy. LVEF of all patients was above 50% at baseline. It dropped below 50% in one patient at half-time and in two patients at after radiation examination (Figure 1). The frequency of pericardial effusions increased (a: none, b: one, c: two). LGE was positive in two cases at baseline, no new LGE occurred during radiation therapy. Native T1-times as well as λ were in the range of reference values at all time points and showed no major change (Table 1).

**Table 1:** Mean course of LVEF, native T1 times and λ

	Baseline	Half-time of radiation therapy	After radiation therapy
LVEF [%]	58 ± 3	56 ± 5	57 ± 7
Native T1 [ms]	966 ± 39	956 ± 14	968 ± 72
λ	0.41 ± 0.04	0.41 ± 0.02	0.42 ± 0.02

## Conclusions

The incidence of pericardial effusion increased during radiation therapy, while CMR tissue analysis failed to identify early myocardial injury in this small number of patients. The LVEF changed in some individuals. A larger trail and subgroup analysis is needed for further differentiation.

